# Novel Diagnostic Model for the Deficient and Excess Pulse Qualities

**DOI:** 10.1155/2012/563958

**Published:** 2011-09-05

**Authors:** Jaeuk U. Kim, Young Ju Jeon, Young-Min Kim, Hae Jung Lee, Jong Yeol Kim

**Affiliations:** Division of Constitutional Medicine Research, Korea Institute of Oriental Medicine, Daejeon 305-811, Republic of Korea

## Abstract

The deficient and excess pulse qualities (DEPs) are the two representatives of the deficiency and excess syndromes, respectively. Despite its importance in the objectification of pulse diagnosis, a reliable classification model for the DEPs has not been reported to date. In this work, we propose a classification method for the DEPs based on a clinical study. First, through factor analysis and Fisher's discriminant analysis, we show that all the pulse amplitudes obtained at various applied pressures at Chon, Gwan, and Cheok contribute on equal orders of magnitude in the determination of the DEPs. Then, we discuss that the pulse pressure or the average pulse amplitude is appropriate for describing the collective behaviors of the pulse amplitudes and a simple and reliable classification can be constructed from either quantity. Finally, we propose an enhanced classification model that combines the two complementary variables sequentially.

## 1. Introduction

Pulse wave is a pressure wave propagating through the arterial system, generated by the periodic contraction and relaxation of the heart, and its characteristics are influenced by the compliance of the vascular system, blood viscosity, and the functions of major organs. By diagnosing the pulse, trained practitioners can gather elaborate physiological and pathological information on the cardiovascular system, organ functions, patients' constitution, emotional conditions, behavioral patterns, and previous illness, as well as body's homeostatic balance [1–5]. 

Pulse diagnosis has been considered a core component of diagnostics in Oriental medicine for thousands of years. In contemporary Oriental medicine, pulse diagnosis is made dominantly at three adjacent positions along the radial artery in both wrists. A palpation position called Gwan is located on the radial artery closest to the styloid process. Chon is about 10 mm distal from Gwan and Cheok about 10 to 15 mm proximal from Gwan [6]. To diagnose the pulse, an Oriental medical doctor (OMD) places the index, middle, and ring fingers, respectively, at Chon, Gwan, and Cheok and applies varying pressure simultaneously or sequentially to determine the pulse qualities.

A recent survey indicates that about 22% of OMDs rely on pulse diagnosis as the primary diagnostic method, which is next to the inquiry (38%) and observation (27%). About 71% of the survey participants asserted that pulse diagnosis was in their diagnoses [7]. Despite its importance and frequent use in clinics, pulse diagnosis has been criticized for the lack of scientific evidence and for the manual palpation and subjective interpretation of pulse qualities. To provide sound scientific evidence and to overcome the experiential boundary of pulse diagnosis, it is essential to develop objective techniques with standardized protocols to obtain pulse signals, and interpret them into pulse qualities defined in terms of a few measureable physical parameters [1, 8, 9].

With advances in fabrication technology for pulse-taking devices [10–12], progress has been made on the quantification and objectification of pulse diagnosis. The physiological characteristics of the pulse at the three aforementioned palpation positions have been shown to differ, which implies that the pulse at each position conveys different clinical information [10]. According to the theory of correspondence between palpation positions and organs, the pulse at the left Gwan conveys the heart functioning [1]. Based on this theory, Huang et al. studied the characteristics of the pulse at the left Gwan and reported that the spectral energy of the pulse in the fourth to sixth harmonics was overly damped in palpitation patients compared to normal subjects [11]. On the other hand, Liu et al. found that, in the pulse measured at the left Chon, Zen meditation induces more elastic pulse waveforms which might indicate improved performance of the cardiovascular system [12]. A report asserted that heat stress reduces the radial augmentation index (AIr) and cold stress increases AIr [13]. Recently, several publications report on the technical improvement of signal processing for the pulse waveform analysis [14–18]. 

A pulse analyzer may replace the OMDs' pulse diagnosis by fingers if it is capable of analyzing the characteristics of the pulse in terms of fundamental physical parameters such as depth, width, length, force, rhythm, contour, speed, and rate [1, 8]. For this purpose, it requires acquisition of the pulse waveform at different hold-down pressures (equivalently, applied pressures), containing two-dimensional spatial distribution of the pulse amplitude along and across the axis of the radial artery. So far, most works on the radial pulse are limited to pattern classification and feature extractions of the pulse waveform obtained at the optimal applied pressure, aiming to distinguish abnormal pulses from normal pulses. As such, to develop desirable pulse analyzers, more extensive studies are needed. 

There are proposals on how to interpret classical pulse qualities in terms of machine appropriate physical parameters [1, 9]. For instance, some researchers attempted to classify a few pulse qualities that can be identified by pattern recognition [19, 20]. Particularly, Zhang et al. developed two effective pattern classification algorithms to distinguish five different pulse patterns of moderate, smooth, taut, hollow, and unsmooth pulses. 

More recently, a novel diagnostic algorithm to distinguish a deep-lying pulse (sunken pulse) from a superficial pulse (floating pulse) was proposed and validated clinically by the authors [21, 22]. For this purpose, we introduced a normalized coefficient that changes monotonically from 0 to 1; as the pulse amplitude becomes larger at heavy-applied pressures compared to light-applied pressures, the coefficient lies closer to 1 [21]. The floating pulse and sunken pulse are the two pulse qualities representing the pulse depth and they belong to the four principal pulses together with the rapid pulse and slow pulse. Therefore, development of an effective algorithm which classifies a pulse according to its depth is a major achievement.

Another principal pulse parameter is the pulse force or equivalently the pulse power. In this work, we study the pulse classification method according to its force or power. Strictly speaking, no pulse quality is defined only in terms of the force of the pulse [1]. However, the pulse force is the most crucial parameter that determines excess/deficient syndromes and is therefore of great clinical importance. Most pulse qualities that are too weak or excessively strong in its pulsation strength are indicative of the deficiency syndrome or the excess syndrome, respectively. In this study, we consider the deficient/excess pulse to be the representative of forceless/forceful pulse qualities [23]. To develop an objective and reliable classification model, firstly, we delineate samples with deficient and excess pulse qualities (DEPs) based on OMDs pulse diagnoses. By using statistical methods, such as factor analysis and Fisher's discriminant analysis, we examine some candidate variables that contribute to the OMDs' conclusions of the DEPs. Finally, we propose a simple but efficient classification model which best explains OMDs' diagnostic results.

## 2. Quantification of the Deficient and Excess Pulse Qualities

**Figure 1:**
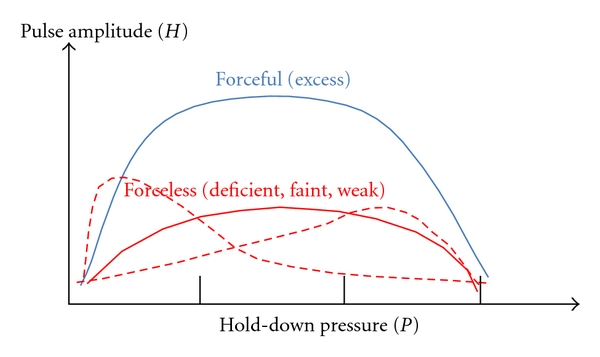
Illustration of forceful (excess) qualities versus forceless (deficient) qualities in the *P*-*H* plane.

Pulse force is a complex parameter determined by the interplay between several variables such as the amplitude of cardiac contraction, volume of blood flow, and the tensile compliance of the arterial wall. A forceful pulse is defined as having large pulse amplitude over a range of the hold-down pressures, while a forceless pulse is defined as one with small pulse amplitude (Figure 1). Forceful pulse qualities include the excess (*Shi*), long (*Chang*), flooding (*Hong*), tight (*Jin*), and wiry (*Xian*) pulses, and forceless qualities include the deficient (*Xu*), weak (*Ruo*), faint (*Wei*), scattered (*San*), and soft (*Ru*) pulses [1]. Excess pulse (*Shi Mai*) and deficient pulse (*Xu Mai*) are the representatives of the forceful pulse qualities and forceless pulse qualities, respectively. 

 The excess pulse is felt strong at all depths from superficial to deep level, felt wide, and it is felt forceful at more than one palpation positions and the pulse is stretched beyond Cheok and/or beyond Chon positions (Figure 1). By combining with other pulse parameters, it can be distinguished from similar pulse qualities. For instance, the flooding pulse has additional properties such as floating and wide, and the wiry pulse to be less forceful and narrower than the excess and flooding pulses. The excess pulse occurs when excess perverse heat is accumulated in the three heaters in the body. Clinically, probable symptoms include insanity, mania, qi pain, yang toxins, vomiting, and other similar symptoms or it may indicate simple accumulation of perverse yang. Depending on the palpation positions, it is likely to indicate food accumulation (Gwan), constipation (Cheok) due to bound heat in the stomach (Gwan) and the intestines (Cheok), and headache, fever, sore throat, stiffness at the root of tongue, or stiffness in the chest and diaphragm (Chon) [2]. 

On the other hand, the deficient pulse lacks pulsation intensity. It is felt either weak through the entire range of pressure, or it is easily perceived with light pressure and ceases to be felt with heavy pressure due to arterial occlusion under heavy hold-down pressure (Figure 1). It can be distinguished from other forceless pulse qualities if other pulse parameters are additionally considered. For instance, the weak pulse is felt at a deeper level. The deficient pulse usually indicates deficiency in both qi and blood. Likely clinical symptoms include lethargy, shortness of breath, spontaneous sweating, pale complexion, low voice, dizziness, and pale tongue [2].

## 3. Subjects and Methods

### 3.1. Study Subjects

The study was approved by the ethics committees of the Korea Institute of Oriental Medicine, and informed written consent for the study was obtained from all subjects prior to study entry (I0903-01-02). Out of hundreds of healthy volunteers in their 20s with no vascular deformity on the radial artery, one hundred subjects were chosen by pairs of OMDs as appropriate candidates for diagnosing the pulse either with deficient or excess pulse qualities. The basic physiological data of the subjects are summarized as number or as mean ± SD in Table 1. 

### 3.2. Study Design

**Figure 2:**
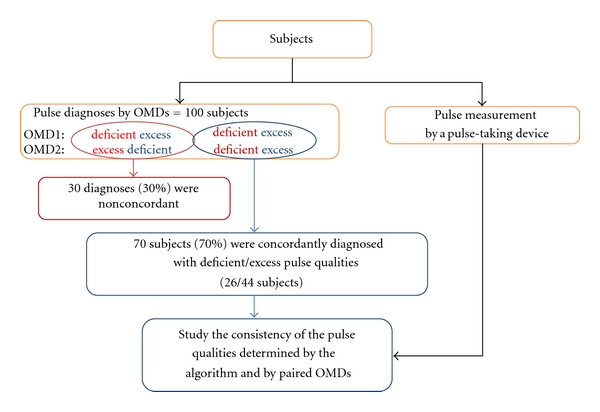
Study design and the outcomes of OMDs' pulse diagnoses.

The study design is shown in Figure 2. On each day of study, according to a given schedule, different pairs of OMDs from an OMD pool with eleven practitioners with more than five years of clinical experience were assigned to make pulse diagnosis. The paired OMDs were asked to independently examine each subject. To avoid confusion when characteristic pulse feelings were different between the left pulse and the right pulse, only the left wrist of each subject was used for pulse diagnosis. Of the 100 subjects chosen by the paired OMDs as appropriate candidates for pulse diagnosis for the DEPs, diagnoses on 70 subjects were concordant between the OMD pairs. Using a pulse-taking device, the pulse waveforms were obtained at the three palpation positions of Chon, Gwan, and Cheok in the left arms. We analyzed the pulse waveforms in the 70 subjects diagnosed either with the deficient pulse or with the excess pulse and attempted to develop a classification model for the DEPs which best explains OMDs' diagnoses.

### 3.3. Pulse Waveform Acquisition

**Figure 3:**
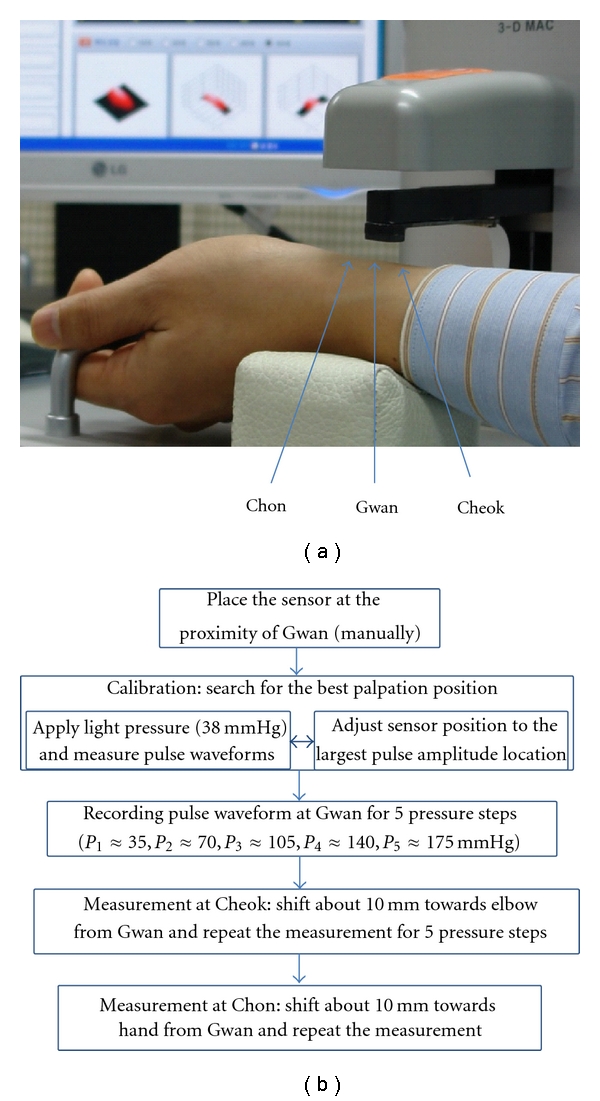
(a) Illustration of a pulse-taking operation by 3D MAC and (b) outline of the automated pulse-taking procedure by 3D MAC.

Pulse waveform was obtained by 3D MAC (Daeyomedi Co., Korea) which was commercially available and approved by the Korea Food and Drug Administration (KFDA). The 3D MAC operates with the applanation tonometry method to apply pressure and acquire pulse waveforms at the traditional palpation positions of Chon, Gwan, and Cheok (Figure 3). The device uses a motor-actuated pressure sensor, which contains 5 sensing elements arrayed crosswise within 10 × 10 mm^2^. Each sensing element is a piezo-resistive sensor of size about 2 × 3 mm^2^.  Figure 3(a) illustrates a pulse-taking operation, with its operation procedure outlined in Figure 3(b). As shown in Figure 3(b), after an operator places the sensor at the proximity of Gwan, by an automated algorithm, the device fine-tunes the sensor location and measures pulse waveforms with varying applied pressures at five discrete pressure steps, for example, at *P*_1_, *P*_2_, *P*_3_, *P*_4_, and *P*_5_. Each pressure step is maintained constant for five seconds. After measuring the pulse waveform at Gwan, the sensor moves towards Cheok and Chon to repeat the measurement. In this study, the hold-down pressure at each pressure step was maintained at *P*_1_ = 37 ± 4, *P*_2_ = 73 ± 5, *P*_3_ = 109 ± 5, *P*_4_ = 143 ± 7, and *P*_5_ = 184 ± 7 mmHg on average (±standard deviation) and the device estimated that Cheok and Chon were located about 10 mm away from Gwan along the radial artery which were comparable to the average palpation positions by OMDs for pulse diagnosis (Figure 3) [6]. Pulse pressures measured by the 3D MAC were in the acceptable range of repeatability (within about 11% of coefficient of variation).

### 3.4. Signal Processing

**Figure 4:**
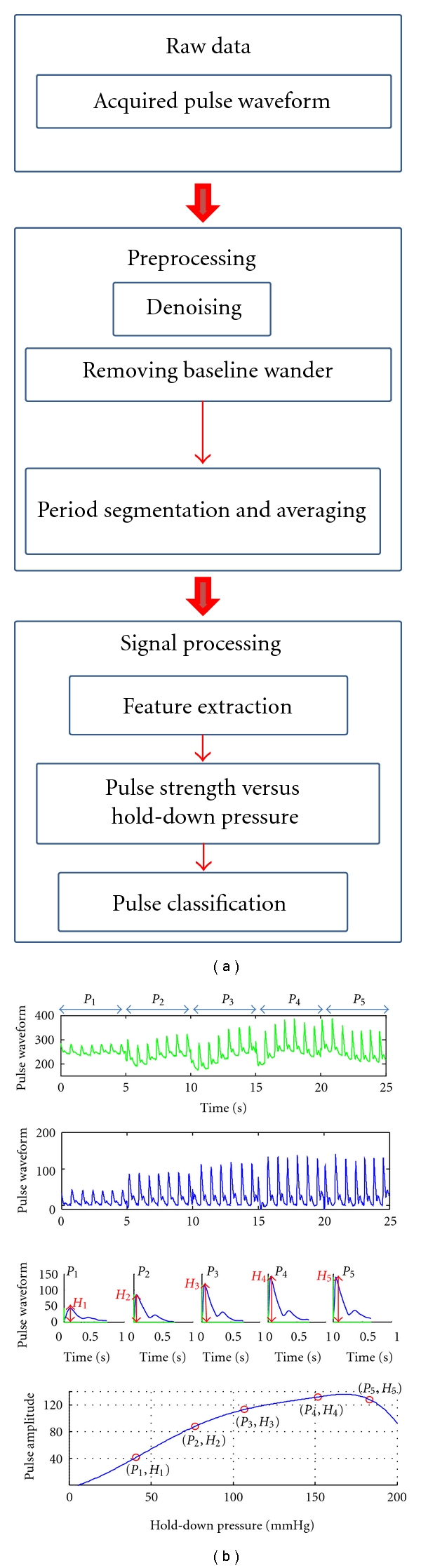
Outline of data manipulation from raw data (top panel) to feature extraction (bottom panel).

Figure 4 illustrates data processing towards the pulse classification algorithm. Raw data (top panel) contained noise due to breathing, uncontrolled movement of subject's arm, and so forth. Therefore, it required preprocessing to remove noise and to align baseline followed by period segmentation and averaging (second panel). We used a nearest neighbor interpolation technique to remove abrupt signal variation, and the 5th order polynomial approximation and subsequent spline interpolation to remove baseline wander. Finally, as outlined in the bottom panel in Figure 4, for each applied pressure step *P*_*j*_ (*j* = 1, 2, 3, 4, and 5), we calculated the maximum amplitude of the pulse waveform *H*_*j*_ which we call the pulse amplitude at given pressure step *P*_*j*_, which would be used to find the classification method.

### 3.5. Pulse Amplitudes

**Figure 5:**
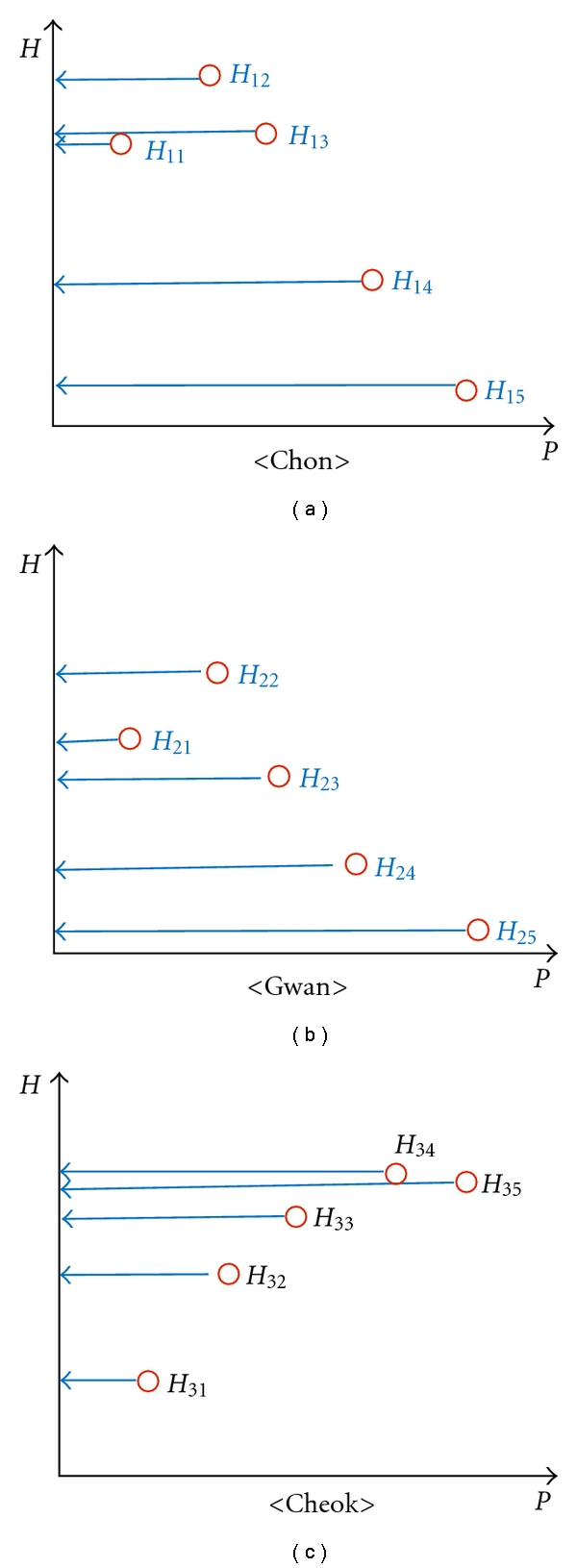
An example of pulse amplitude (*H*) versus applied pressure (*P*) at Chon, Gwan, and Cheok. To distinguish pulse amplitudes at different palpation positions, locational label was newly introduced to indicate that *H*_*ij*_ is the pulse amplitude at *i*th (*i* = 1, 2, and 3) palpation position and at *j*th (*j* = 1, 2, 3, 4, and 5) pressure step.

In previous subsections, we discussed discrete pressure steps and pulse amplitudes (*P*_*j*_, *H*_*j*_) without distinguishing different palpation positions. As will be discussed below, however, to determine the DEPs, OMDs use all the pulse amplitudes at Chon, Gwan, and Cheok. To differentiate between different palpation positions, as in Figure 5, we introduce location label so that *H*_*ij*_ indicates the pulse amplitude at *i*th palpation position (*i* = 1, 2, and 3 for Chon, Gwan, and Cheok, resp.) and at *j*th pressure step (*j* = 1, 2, 3, 4, and 5 from the lightest to the heaviest applied pressure). 

There are some pulse quantities derived from the pulse amplitudes that are potentially relevant in determining the DEPs. The first such quantity is the pulse pressure (PP). The PP, which is known to be an important indicator in predicting coronary heart disease particularly in the middle-aged and the elderly [24], is defined as the difference between systolic blood pressure and diastolic blood pressure in a cardiac cycle. The PP is equivalent to the maximum amplitude among the pulse amplitudes at various applied pressure steps, that is, PP_*i*_ = *H*_*i*_^max ^ ≈ max (*H*_*i*1_, *H*_*i*2_, *H*_*i*3_, *H*_*i*4_, *H*_*i*5_), where max  (⋯) returns the largest value among (⋯) [25]. The average pulse pressure over Chon, Gwan, and Cheok is then given by 〈PP_*i*_〉 = 〈*H*_*i*_^max ^〉 ≈ (*H*_1_^max^ + *H*_2_^max^ + *H*_3_^max^)/3. There may be other pulse quantities to be used relevantly in determining the DEPs. Such a quantity may be the maximum pulse pressure among the three palpation positions, which is defined by PP^max ^ = max (*H*_1_^max^, *H*_2_^max^, *H*_3_^max^). The mean pulse amplitude (MPA) can also contribute in a major way in the determination of the DEPs, MPA_*i*_ = *H*_*i*_^avg^ ≈ (*H*_*i*1_ + *H*_*i*2_ + *H*_*i*3_ + *H*_*i*4_ + *H*_*i*5_)/5. Over the three palpation positions, we define the average and maximum of the MPA_i_ by 〈MPA_*i*_〉 = (*H*_1_^avg^ + *H*_2_^avg^ + *H*_3_^avg^)/3 and MPA^max ^ = max (*H*_1_^avg^, *H*_2_^avg^, *H*_3_^avg^), respectively.

### 3.6. Statistical Method

Statistical analyses were performed by using SPSS version 14.0 (SPSS Inc., USA) and MATLAB version 7. x (Mathworks Inc., USA). Student's *t*-test was performed to compare means of selected continuous variables in the deficient pulse group and excess pulse group. Factor analysis was used to identify groups of highly correlated variables and their relationship to the target variable. We used Fisher's discriminant analysis to determine the best concordance with the diagnostic results of OMDs. To examine the quality of concordance between algorithmic predictions and OMDs' diagnoses, we additionally calculated the Matthews correlation coefficient.

## 4. Results and Discussion

### 4.1. Diagnoses by Paired OMDs

One hundred subjects were selected by paired OMDs to be included in this study. Among them, the diagnoses on 70 subjects (70%) were concordant between paired OMDs, while the diagnoses of the remaining 30 subjects (30%) were divergent. Among the concordant cases, 26 subjects (37%) were diagnosed with deficient pulses and 44 subjects (63%) with excess pulses. 

Table 2 shows that the accuracy (alternatively, concordant diagnoses) between OMD1 and OMD2 was 70% (70 agreements/100 simultaneous diagnoses), and the Matthews correlation coefficient (MCC) was 0.38. The MCC is regarded as one of the best measures of the quality of binary classifications, particularly when the two classes are of very different sizes [26, 27]. An accuracy of about 70% and MCC of about 0.4 are indicative of moderate concordance between OMDs' diagnoses, noting that the diagnoses of Table 2 were not made by any fixed pairs of OMDs, but by cyclically paired OMDs among a pool of 11 OMDs on each day of study.

### 4.2. Characteristics of the Deficient Pulse Group and Excess Pulse Group

**Figure 6:**
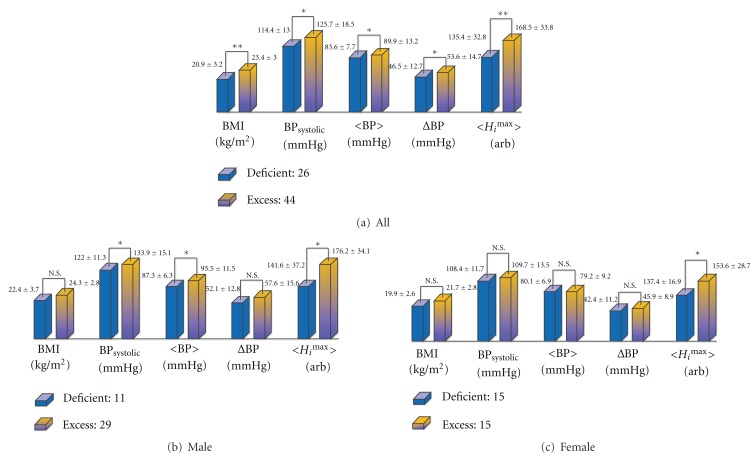
Characteristics of subjects diagnosed with the DEPs stratified by gender; (a) for the entire sample size, (b) for male subjects, and (c) for female subjects. Data presented are the mean ± SD, and the *P* value from Student's two sample *t*-test. *P < 0.05, **P < 0.005; N.S: nonsignificant. Abbreviated: “BP_systolic_” = systolic blood pressure, “〈BP〉” = average blood pressure, “ΔBP” = BP_systolic_ − BP_diastolic_, and “*H*_*i*_^max ^” stands for the maximum pulse amplitude at the *i*th palpation position whose unit was determined by the manufacturer (arb), and “〈*H*_*i*_^max ^〉” is the average of *H*_*i*_^max^ over *i* = 1, 2, and 3.

In total, 70 subjects were concordantly diagnosed with deficient or excess pulses by paired OMDs.  Figure 6 summarizes the means and standard deviations (SD) of some relevant physiological quantities for each pulse group, stratified by gender. In addition, a Student's *t*-test was applied between the two pulse groups. For the entire cohort, BMI, systolic (BP_systole_) average blood pressure 〈BP〉, the difference between systolic and diastolic blood pressure (ΔBP), and 〈*H*_*i*_^max ^〉 were significantly different between the two pulse groups at a significance level of 0.05, while diastolic blood pressure and heart rate remained nonsignificantly different (not shown in the figure). Here, as introduced in the previous section, *H*_*i*_^max^ is the approximate pulse pressure at *i*th palpation position measured by the pulse-taking device (3-D MAC) and 〈*H*_*i*_^max ^〉 is the average over Chon, Gwan, and Cheok in the left wrist [28], while ΔBP is the pulse pressure measured at the right brachial artery by a commercial sphygmomanometer (FT-750(R), Jawon Medical, Korea).

Among the five statistically significant quantities, 〈*H*_*i*_^max ^〉 were the most significantly different between the two pulse groups, which implies that, among the compared quantities, the average pulse pressure over the three palpation positions is the most appropriate choice as the decision parameter for the DEPs. The reduced significance in all variables in each gender is mostly due to reduced sample size; 〈*H*_*i*_^max ^〉 remained significantly different by gender, while such statistical differences were diminished in other variables.

The pulse pressure is known to be different throughout the large artery tree, while the mean arterial pressure remains constant and the diastolic pressure does not change substantially throughout the large artery tree [25]. In addition, the pulse pressures and other pulse parameters at the right and left arms usually show distinctive characters [29]. Therefore, it is reasonable to have discrepancy in the significance level between the pulse pressures at the left radial artery (〈*H*_*i*_^max ^〉) and at the right brachial artery (ΔBP). On the other hand, it has been shown that the BMI is marginally valid in distinguishing the deficient pulse group from the excess pulse group. It implies that an obese individual is more likely to have an excess pulse than a thin individual, which is in agreement with clinical experience.

Since OMDs palpated the radial artery to diagnose the DEPs, among physiological quantities found at various locations along the large artery tree, a properly defined quantity on the radial artery is expected to show the best correlation with OMDs' diagnostic result. Therefore, it is intuitively correct that the pulse pressure measured at the radial artery (〈*H*_*i*_^max ^〉) was the most appropriate parameter in distinguishing the deficient pulse group from the excess pulse group. More importantly, it implies that the pulse pressure 〈*H*_*i*_^max ^〉 or similarly defined pulse quantities on the radial artery contribute significantly to the determination of the DEPs. In the following, to determine which pulse quantities contribute significantly to the OMDs' diagnoses of the DEPs, we perform factor analysis accompanied by Fisher's discriminant analysis.

### 4.3. Factor Analysis

As described previously, we observed that the average pulse pressure 〈*H*_*i*_^max ^〉 is one of the most promising candidates as the decision variable for the DEPs. Since 〈*H*_*i*_^max ^〉 is derived from the pulse amplitudes *H*_*ij*_, it is desirable to scrutinize *H*_*ij*_ to determine which of them are important and in which combination it best explains the decision rule of OMDs' diagnoses of the DEPs. For this purpose, we performed factor analysis of 15 pulse amplitudes from *H*_11_ to *H*_35_. Factor analysis attempts to describe the covariance relationship among many variables in terms of a few underlying latent quantities, called *factors* [30]. It reduces attribute space from a larger number of variables to a smaller number of factors. To find factors, we followed the principal component method. For the rotation of variables, we followed the varimax procedure. Kaiser-Meyer-Olkin (KMO) measure of sampling adequacy was 0.672, and Bartlett's test of sphericity was valid with *P* < 0.01 of significance level, both of which indicate the appropriateness of factor analysis. By factor analysis, we obtained 5 significant factors with eigenvalues larger than one, which accounted for about 80% of the variance as in Table 3. Note that an eigenvalue represents the amount of variance associated with the factor, and therefore only factors with variance greater than one are expected to show better performance than an initial individual variable, that is, *H*_*ij*_, whose variance is normalized to one [30].

As in Table 3, we determined the 5 most relevant factors which accounts for about 80% of the variance, in which all 15 pulse amplitudes contribute once and only once with likely weight; factor loadings are in equal orders of magnitude, ranging between 0.58 and 0.93 [31]. The two most contributing factors account for about 54% of the variance, in which the pulse amplitudes at all applied pressures at Cheok (*H*_31_ to *H*_35_) and the pulse amplitudes at light-applied pressures at Gwan (*H*_21_ and *H*_22_) are almost equally involved with similar factor loadings. In summary, all 15 pulse amplitudes were found to be important in explaining the total variance, and the pulse amplitudes with light-applied pressures and heavy-applied pressures were grouped into different factors, implying possibly different roles in pulse classifications between the two pulse amplitude groups with light-applied pressures and heavy-applied pressures.

### 4.4. Fisher's Discriminant Analysis with the Five Factors Obtained from Factor Analysis

With the five factors listed in Table 3, we continued to perform Fisher's discriminant analysis to determine a discriminant function for the DEPs with reference to the OMDs' diagnoses [30]. The value of Box's *M* test was 12.29 (*P* value = 0.737), which satisfies the assumption of equal variance. The discriminant function was found to be significant with a Wilks' Lambda of 0.793 (*P* value = 0.010). The standardized canonical discriminant function coefficients for the 5 factors are listed in Table 4, and the classification results are shown in Table 5. The magnitude of a coefficient indicates the contribution weight of the given factor. Table 4 shows that factors *f*_1_, *f*_4_, and *f*_5_ contribute almost equally, and the contribution of *f*_2_ and *f*_3_ are, respectively, about a half and a fifth of the major factors which are in the same order of magnitude. It is worth mentioning that no factor alone governs the behavior of the discriminant function but all the participating factors contribute evenly.

By combining the results in Tables 3 and 4, we notice that all the pulse amplitudes at light- and heavy-applied pressures at Chon, Gwan, and Cheok contribute on equal orders of magnitude to the classification of the DEPs. More rigorously speaking, the most contributing factors of *f*_1_, *f*_4_, and *f*_5_ are comprised of the pulse amplitudes at light-applied pressures at Chon (*H*_11_ and *H*_12_) and the pulse amplitudes at heavy-applied pressures at Gwan (*H*_23_, *H*_24_, and *H*_25_) and Cheok (*H*_33_, *H*_34_, and *H*_35_). It is clinically known that the optimal pulse depth at which the pulse is felt strongest is shallower at Chon than at Gwan or Cheok. Applying this clinical knowledge, the pulse pressure and the factors of *f*_1_, *f*_4_, and *f*_5_ are well correlated, which implies that the pulse pressure may be an appropriate quantity for the classification of the DEPs, which is one of our target variable to be discussed below. 

We applied the coefficients in Table 4 to Fisher's discriminant function and obtained the classification result in Table 5. The accuracy of the classification for the entire data set is 72.9% with the Matthews correlation coefficient of 0.46. We repeated the leave-one-out cross-validation test and obtained the accuracy of 61.4% (MCC = 0.24). Reduced accuracy of about 11% in the cross-validation test indicates that the generated discriminant function overfits the training set, which opens the possibility for more efficient classification with less variables.

### 4.5. Fisher's Discriminant Analysis with the Representative Pulse Quantities at Each Palpation Position

By factor analysis, we found that all the pulse amplitudes at various levels of applied pressures at the three palpation positions contributed with similar weight in the determination of the DEPs. OMDs rely mostly on the pulse force to determine the DEPs. A pulse may be considered forceful if either its maximum amplitude is large or the average amplitude over various applied pressures is large. The former is the pulse pressure and the latter is the mean pulse amplitude. In search of a simple form for the discriminant function with improved accuracy compared to the result using factor analysis, the two most relevant quantities are thought to be the pulse pressure PP_*i*_ = *H*_*i*_^max^ and the mean pulse amplitude MPA_*i*_ = *H*_*i*_^avg^ at each (*i*th) palpation position, whose detailed expressions in terms of *H*_*ij*_ are introduced in Section 3.5. With the pulse pressures and the mean pulse amplitudes at the three palpation positions, in the following, we apply Fisher's discriminant analysis to determine an efficient classification model.

In the canonical correlation analysis, the coefficients of the standardized canonical discriminant function for *H*_1_^max^, *H*_2_^max^, and *H*_3_^max^ were, respectively, given by 0.505, 0.282, and 0.555, which indicates that the three pulse pressures contribute to the discriminant function on equal orders of magnitude. The classification result by the linear discriminant function using the three pulse pressures is summarized in Table 6. The accuracy of the classification for the entire data set is 78.6%, and the Matthews correlation coefficient is 0.56. By a cross-validation test, we obtained the accuracy of 75.7% (MCC = 0.51).

By repeating the canonical correlation analysis using *H*_1_^avg^, *H*_2_^avg^, and *H*_3_^avg^, we again find that the 3 involved variables contribute to the discriminant function on equal orders of magnitude (the standardized canonical discriminant function coefficients are given by 0.248, 0.533, and 0.459 resp. for *H*_1_^avg^, *H*_2_^avg^, and *H*_3_^avg^). The accuracy of the classification for the entire data set was 75.7% (MCC = 0.51), and the cross-validated classification accuracy was 68.6% (MCC = 0.36). Using both the maxima and averaged variables together, we obtained a classification accuracy of 72.9% (MCC = 0.45) for the entire data set and 65.7% (MCC = 0.30) by the cross-validation test, which is worse than the three pulse pressures or the three averaged variables separately. In conclusion, the three pulse pressures *H*_1_^max^, *H*_2_^max^, and *H*_3_^max^ were relevantly contributing to the pulse decisions for the DEPs, while additional or independent participation of the mean pulse amplitudes did not improve the concordance rate with OMDs' pulse decisions.

### 4.6. Diagnostic Model with the Representative Pulse Quantities over All the Palpation Positions

Let us further reduce the number of pulse variables by taking the maximum or the average of pulse quantities *H*_*i*_^max^ and *H*_*i*_^avg^ over the three palpation positions. This reduction procedure is based on the following reason. When a pulse is considered forceful over the three palpation positions, it may indicate that the maximum of a pulse quantity over the three palpation positions is large or that the average over the palpation positions is large. 

Following this procedure, we obtain four pulse quantities, such as 〈PP_*i*_〉, PP_*i*_^max^, 〈MPA_*i*_〉, and MPA_*i*_^max^ (see Section 3.5 for detailed relations with *H*_*ij*_'s), as competing variables for the pulse classification. By repeating Fisher's discriminant analysis, as summarized in Table 7, we obtained good accuracy and Matthews correlation coefficient with the average pulse pressure (〈PP_*i*_〉) or with the average of the MPAs (〈MPA_*i*_〉), while less accurate classification was obtained with the maximum pulse pressure (PP_*i*_^max^) or with the maximum of the MPAs. The combination of the four variables does not increase the accuracy of the discrimination, but rather reduces it due to overfitting.

We can improve the accuracy beyond the limit of the linear discriminant analysis by a mixed-variable classification model, where two competing variables participate sequentially in the decision process in complementary manner. To build an appropriate mixed-variable classification model, we review a simplest linear discriminant function with one participating variable; with 〈PP_*i*_〉, the rule is given by

(1) $$\begin{array}{c} {}[\text{If}\;\langle {\text{PP}}_{i}\rangle \geq \alpha ,\;\text{then}\;\text{it}\;\text{is}\;\text{an}\;\text{excess}\;\text{pulse}\;\text{quality},\;\;\\ \;\;\;\;\text{while}\;\text{if}\;\langle {\text{PP}}_{i}\rangle <\beta ,\;\text{then}\;\text{it}\;\text{is}\;\text{a}\;\text{deficient}\;\text{pulse}\;\text{quality}], \end{array}$$

where parameters *α* and *β* are the criteria used for decision on the DEPs and they satisfy *α* ≥ *β*. When *α* = *β*, the accuracy and the MCC are as summarized in Table 7. In this simple discriminant analysis, there exists an intermediate regime between or near the criterion parameters *α* and *β*, where the concordance of the pulse decisions with the OMDs' decisions becomes abruptly poor; in the decision making for 〈PP_*i*_〉 far above *α* as an excess pulse or for 〈PP_*i*_〉 far below *β* as a deficient pulse, the concordance is usually very good.

**Figure 7:**
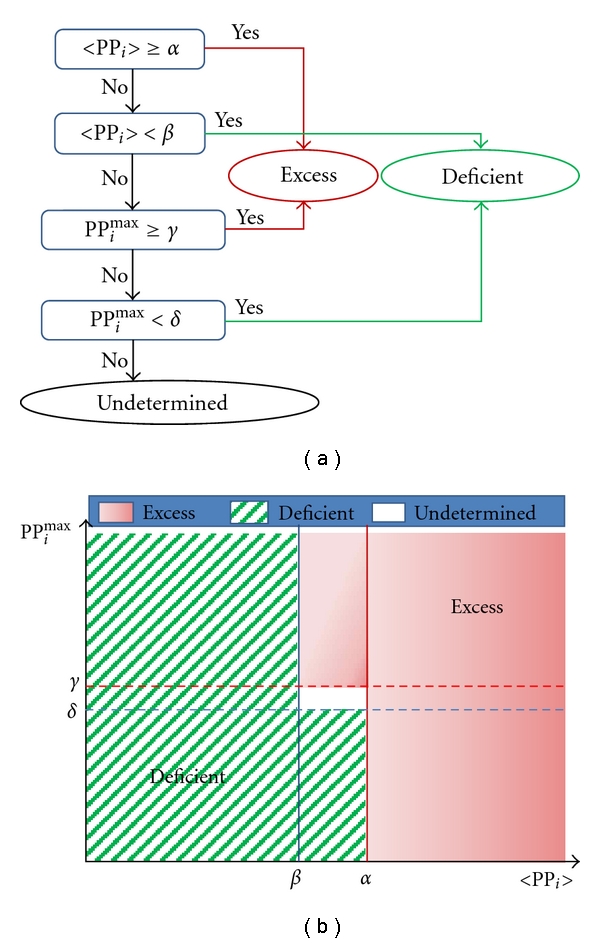
A mixed-variable diagnostic method for the DEPs. (a) flowchart for the classification, and (b) classification regimes for the DEPs.

An improved accuracy is possible by substituting the intermediate regime of discordance (the regime between *α* and *β*) with another candidate variable such as PP_*i*_^max^ or MPA_*i*_^max^. Despite the correlation between these variables is high (Pearson correlation coefficient ≥ 0.8), the intermediate regimes of discordance between themselves are slightly off-resonance. It gives an opportunity to improve accuracy by letting the secondary variable intervene in the intermediate regime of discordance of the primary variable. The standard classification procedure for this mixed-variable diagnostic model is outlined in Figure 7. It yielded an accuracy of 80.0% and MCC of 0.57 (after standardization, the criterion parameters were, resp., given by *α* = −0.216, *β* = −0.572, and *γ* = *δ* = −0.314), which was about 5.7% increase in the accuracy and 10% increase in the MCC compared to the original discriminant function with 〈PP_*i*_〉 alone. Applying 〈MPA_*i*_〉 and 〈PP_*i*_^max ^〉 as the two variables in the mixed-variable diagnostic model, it yielded similar improvement in performance (accuracy of 80%). The increased accuracy of the mixed-variable discriminant model implies that the average and maximum pulse amplitudes over the palpation positions are complementary to each other in OMDs' pulse decisions.

## 5. Conclusions

For the objectification and standardization of pulse diagnosis, reliable classification methods for the principal pulse qualities are urged to be developed. The deficient and excess pulse qualities (DEPs) are clinically important as they are the indicators representing the deficiency syndrome and the excess syndrome, respectively. In this work, we proposed a classification method for the DEPs. For this purpose, we conducted a clinical test and selected 70 subjects in their 20s either with the deficient pulse (26 samples) or with the excess pulse (44 samples), by concordant diagnoses between paired OMDs. By a Student's *t*-test, among several candidate quantities such as the BMI or systolic/diastolic blood pressures measured at the brachial artery, we confirmed that the average pulse pressure defined over the three palpation positions was the most appropriate quantity in distinguishing the excess pulse group from the deficient pulse group. We continued to apply factor analysis and Fisher's discriminant analysis and found that all the pulse amplitudes obtained at various applied pressures at the three palpation positions contributed relevantly to the diagnosis of the DEPs. 

Next, we showed that either of the pulse pressure or the average pulse amplitude yielded as good accuracy as the original pulse amplitudes. It reflects that the diagnoses of the DEPs by OMDs rely mostly on the pulse force, as either of the two quantities is appropriate in representing the pulse force and good at describing the collective behavior of the original pulse amplitudes. Finally, we proposed a mixed-variable classification model, in which two complementary variables, for example, either two of the maximum or average of the pulse pressures, or the maximum or average of the mean pulse amplitudes, acted over the three palpation positions, were used sequentially to increase the classification accuracy in a reasonable degree. This study will contribute to the objectification and standardization of pulse diagnosis.

## Figures and Tables

**Table 1:** Basic physiological data of the subjects.

Characteristic (unit)	Number or mean ± SD
Number (*n*)	100 (male = 50, female = 50)
Age (yr)	23.8 ± 2.4
Height (cm)	168.0 ± 8.1
Weight (kg)	63.8 ± 12.3
BMI (kg /m^2^)	22.4 ± 3.1
Systolic/diastolic blood pressure (mmHg)	119.2/68.5 ± 17.4/13.0

**Table 2:** Concordance of the pulse diagnoses between paired OMDs.

		OMD1		
		Deficient	Excess	Total
OMD2	Deficient	26	17	43
	Excess	13	44	57
	Total	39	61	100

**Table 3:** Factor analysis of the 15 pulse amplitudes.

Factor	Variable	Factor loading	Eigenvalue	% of variance
*f*_1_	*H*_33_	0.812	4.987	33.245
	*H*_34_	0.928		
	*H*_35_	0.826		

*f*_2_	*H*_21_	0.770	3.190	21.267
	*H*_22_	0.686		
	*H*_31_	0.766		
	*H*_32_	0.789		

*f*_3_	*H*_13_	0.741	1.430	9.533
	*H*_14_	0.887		
	*H*_15_	0.713		

*f*_4_	*H*_23_	0.575	1.252	8.347
	*H*_24_	0.900		
	*H*_25_	0.913		

*f*_5_	*H*_11_	0.818	1.214	8.091
	*H*_12_	0.906		

**Table 4:** The standardized canonical discriminant function coefficients for the five factors in Table 3.

	*f*_1_	*f*_2_	*f*_3_	*f*_4_	*f*_5_
Coefficient	0.575	0.219	0.091	0.538	0.489

**Table 5:** Pulse classification with the 5 factors obtained from factor analysis into the DEPs.

		Classification by discriminant function		Total (*N*)
		Deficient pulse	Excess pulse	
OMD diagnosis	Deficient Excess	20 (76.9%)	6 (23.1%)	26
		13 (29.5%)	31 (70.5%)	44
Cross-validation	Deficient Excess	17 (65.4%)	9 (34.6%)	26
		18 (40.9%)	26 (59.1%)	44

**Table 6:** Pulse classification result by *H*_1_^*max*^, *H*_2_^*max*^, and *H*_3_^*max*^ into the DEPs.

		Classification by discriminant function		Total (*N*)
		Deficient pulse	Excess pulse	
OMD diagnosis	Deficient Excess	21 (80.8%)	5 (19.2%)	26
		10 (22.7%)	34 (77.3%)	44
Cross-validation	Deficient Excess	20 (76.9%)	6 (23.1%)	26
		11 (25.0%)	33 (75.0%)	44

**Table 7:** Fisher's discriminant analysis with 〈*PP*_*i*_〉, *PP*_*i*_^*max*^, 〈*MPA*_*i*_〉, and *MPA*_*i*_^*max*^.

	〈PP_*i*_〉		PP_*i*_^max ^		〈MPA_*i*_〉		MPA_*i*_^max ^		Sum	
	Accuracy	MCC	Accuracy	MCC	Accuracy	MCC	Accuracy	MCC	Accuracy	MCC
Entire data	74.3%	0.47	64.3%	0.30	74.3%	0.48	70.0%	0.39	65.7%	0.32
Cross-validation	72.9%	0.45	64.3%	0.30	74.3%	0.48	70.0%	0.39	62.9%	0.26
